# Repurposing mechanistic insight of PDE-5 inhibitor in cancer chemoprevention through mitochondrial-oxidative stress intervention and blockade of DuCLOX signalling

**DOI:** 10.1186/s12885-019-6152-9

**Published:** 2019-10-24

**Authors:** Manjari Singh, Sweta Kasna, Subhadeep Roy, Sara Aldosary, Abdulaziz S. Saeedan, Mohd. Nazam Ansari, Gaurav Kaithwas

**Affiliations:** 1 Department of Pharmaceutical Sciences, Babasaheb Bhimrao Ambedkar University, (A Central University), Vidya Vihar, Raebareli road, Lucknow, UP 226 025 India; 2 Department of Pharmaceutical Sciences, King Faisal University, Al-Ahsa, Saudi Arabia; 3 Department of Pharmacology, College of Pharmacy, Prince Sattam Bin Abdulaziz University, Al-Kharj, Kingdom of Saudi Arabia

**Keywords:** Tadalafil, ER+ mammary gland cancer, Mitochondrial stress, PDE-5 inhibitor, N-methyl-n-nitrosourea, DuCLOX

## Abstract

**Background:**

This study evaluates the anti-cancer effects of Tadalafil (potent PDE-5 inhibitor) in female albino wistar rats against n-methyl n-nitrosourea induced mammary gland carcinogenesis.

**Methods:**

The animals were selected and randomly divided among four groups and each group contains six animals per group. The animal tissue and serum samples were evaluated for the presence of antioxidant parameters and the cellular morphology was studied using carminic staining, haematoxylin staining and scanning electron microscopy followed by immunoblotting analysis.

**Results:**

On the grounds of hemodynamic recordings and morphology, n-methyl n-nitrosourea treated group showed distorted changes along with distorted morphological parameters. For morphological analysis, the mammary gland tissues were evaluated using scanning electron microscopy, whole mount carmine staining, haematoxylin and eosin staining. The serum samples were evaluated for the evaluation of oxidative stress markers and inflammatory markers. The level of caspase 3 and 8 were also evaluated for the estimation of apoptosis. The fatty acid profiling of mammary gland tissue was evaluated using fatty acid methyl esters formation. The mitochondrial mediated apoptosis and inflammatory markers were evaluated using immunoblotting assay.

**Conclusion:**

The results confirm that Tadalafil treatment restored all the biological markers to the normal and its involvement in mitochondrial mediated death apoptosis pathway along with inhibition of inflammatory markers.

## Background

Distinct metastasis and resistance development by self-renewing nascent cancer cells in response with the chemotherapy drug regimen and simultaneous radiations are still a main obstacle in cancer treatment for patients nowadays [1]. Tadalafil is a known phosphodiesterase-5 (PDE-5) inhibitor, which have anti-cancer effects in various types of cancer [2]. In the search of molecularly targeted compounds that have the structure similarity with the known anti-cancerous drugs like viscristine, vinblastin, vindesine, vinorelbine, leuprolide and geserelin got tremendous attention due to the expression and regulation of the important signalling pathways [3]. In the last decade, Tadalafil used to inhibit the myeloid derived suppressor cells (MDSCs) and restore the T cells in cancer patients as compared to the normal peoples [4]. PDE-5, PDE-6, PDE-9 are the predominant active isoform of ubiquitously distributed metallohydrolases constitute of 11 distinct gene families, which initiate the sequential lcleavage of cyclic adenosine monophosphate (cAMP) and cyclic guanosine monophosphate (cGMP) into their intermediate inactive 50 derivatives and 5-GMP. It ultimately regulating the amplitude and duration of their intracellular downstream signaling mechanism [5]. This inhibition of cGMP conversion leads to activate protein kinase G and map kinase pathway of apoptosis and also simultaneously affect cancer cell growth and adhesion, mitochondrial energy homeostasis, neuronal signaling and muscle relaxation in systemic vasculature, prostate, heart, brain, lungs and platelets [6]. In previous literature, it was reported that PDE-5 inhibition produced caspase dependent apoptosis of B-cell chronic lymphocytic leukemic cells [7]. Various preliminarily studies also demonstrated that PDE-5 inhibitors can induce apoptosis by activation of cGMP and nitric oxide (NO) in cancerous cell [8]. In recent findings, indole derivatives have the potency to treat mammary gland cancer and Tadalafil also have the indole moiety in its structure, which ultimately reveals its molecular mechanism [9]. It was reported that the expression of PDE-5 was significantly high in the malignant breast tumors as compare with normal breast tissues and benign tumors [10]. Consistently increased PDE-5 expression has also been reported in a variety of mammary gland cancer cell lines (MCF-7, T47D, HTB-26, MDA-MB-231), which gives the rationale to assess the anticancer effects of PDE-5 inhibitors in carcinogen induced animal model and check the involvement of reactive oxygen species (ROS), death receptor and mitochondrial signalling as part of the combinatorial apoptosis mechanism. Based on the previous literature, we hypothesize to evaluate the effect of tadalafil upon carcinogen induced ER+ mammary gland carcinoma in vivo model.

## Methods

### Drugs and chemicals

Tadalafil (API) was procured from the Sanofi Aventis as a gift sample followed by FTIR analysis to establish its purity. N-methyl-n-nitrosourea (MNU) (Sigma-Aldrich, N1517) was procured from Sigma Life Science Aldrich Co. 3050 Spruce Street, St. Louis, USA. Other chemicals were purchased from Himedia Pvt. Ltd., Sigma Aldrich and Amresco.

### In vivo study

The study protocol was approved from CPCSEA guidelines for laboratory Animals and Ethics, Government of India (IAEC/SHIATS/PA16III/SSPG19). Female albino wistar rats of 100-120 g were used for this study and collected from the central animal house facility of Sam Higginbottom University of Agriculture, Technology and Sciences, Naini Allahabad, India. All animals were randomized among four groups having eight animals each. Carcinogenesis was induced by single tail vein injection of MNU on day 1^st^in each animal. MNU was dissolved immediately before use in glacial acetic acid and water (pH 4.5–5). The experimental groups were randomized as follows: group I (normal control, saline 3 ml/kg, p.o.; group II (toxic control, MNU 47 mg/kg, i.v.); group III (Tadalafil 2 mg/kg, p.o. + MNU 47 mg/kg, i.v.) and group IV (Tadalafil 4 mg/kg, p.o. + MNU 47 mg/kg, i.v). Tadalafil was administered from 7th to 110th day at the dose specified above. At the end of study, animals were sacrificed for the collection of mammary gland tissue under ketamine hydrochloride (100 mg/kg, i.m.) and diazepam (5 mg/kg, i.m.) combination as anaesthesia.

All the animal experimental procedures follow the ARRIVE guidelines.

### Changes in hemodynamic recordings

The hemodynamic changes were recorded using the Lab Chart Pro-8 (AD Instruments, Australia) instrument according to the methods elaborated by us previously [11, 12].

### Effect of Tadalafil upon morphological changes

#### Whole mount alum stain

Rats were sacrificed after completions of study and forth mammary gland tissues were excised and fixed for a minimum of 2 days in Carnoy’s fixative solution followed by staining with carmine solution (1 g carmine and 2.5 g aluminum potassium sulfate in 500 ml water). The fixed glands were hydrated with 90, 70, 35 and 15% ethyl alcohol and then rinsed with water for 5 min. The glands were then dehydrated progressively in 35, 70, 95 and 100% ethanol, dipped in xylene for 2 days and mounted on glass slides. The sections were examined for the identification of angiogenesis [13, 14].

#### Histopathological studies

Thin section (5 μm) of mammary glands tissue was cut using microtome and fixed overnight in 10% hank’s balanced salt solution (HBSS) buffered formalin which further used for representative gross morphological and histological features of haematoxylin and eosin (H&E) stained paraffin sections of the mammary glands tissue [15, 16].

#### Scanning electron microscopy (SEM)

Sodium cacodylate and HBSS buffer based HCl collagenase hyaluronic acid enzymatic digestion method was used for preparation of tissue before SEM analysis. The entire methodology was performed according to methods elaborated by us previously [17]. The sample was dried with increasing grade of acetone (70, 80, 90 and 100%). All the acetone specimens were dehydrated and replaced by exchange of soak/flash liquid CO_2_ fluids with ethanol or acetone by critical point method (31 °C and 74 bar). Samples were further processed by platinum coating, which has been examined under SEM(X1000) (JEOL JSM-6490LV) [15, 18].

### Effect of Tadalafil upon oxidative stress markers

The antioxidant parameters were evaluated using tissue homogenate (10%w/v). The homogenate was prepared in 0.15 M KCl and the same was centrifuged for 15 min at 4 °C. In thiobarbituric acid reactive substances (TBARs), malondialdehyde (MDA) forms a 1:2 adduct with TBAR and produce the reaction which can be measured by spectrophotometer at 540 nm. For superoxide dismutase (SOD), the inhibition of pyrogallol auto-oxidation was measured. The absorbance was measured at 420 nm. Catalase catalyzes the decomposition of hydrogen peroxide to water and oxygen. The absorbance was measured at 250 nm. Glutathione was measured using Ellamanis reagent (DTNB), when DTNB reacts with GSH then 5 thionitrobenzoic acid (TNB) and GS-TNB was formed. The absorbance was measured at 410 nm [19, 20].

### Serum NO level

For NO estimation, same proportion of serum isolated from animal blood and Griess reagent were mixed and incubated at 37 °C for 30 min. The reaction was proceeded at room temperature, and the absorbance was read at 540 nm using UV-visible spectrophotometer (Cary 60, Agilent Technologies, CA, 95051, US). The standard curve was plot using serial dilutions of sodium nitrite [21].

### Estimation of serum hydrogen sulphide (H_2_S) level

For H_2_S estimation, 500 μl of tissue supernatant and 75 μl of serum sample was added to the premixed zinc acetate (1%w/v) solution, respectively. The concentration of H_2_S in the serum sample was measured using the method elaborated by us previously. The absorbance was read spectrophotometrically at 670 nm [22].

### Cyclooxygenase (COX) and lipoxygenase (LOX) estimation

The COX and LOX were estimated in the rat serum sample followed by the method elaborated elsewhere [23].

### Caspase3 and caspase8 estimation

96-well amber colored plate has been used for the colorimetric identification of caspase-3/8. The estimation was performed on the basis of methodology elaborated by us previously [24].

### Gas chromatographic (GC) analysis of mammary gland tissue

The fatty acid methyl esters (FAME) analysis was performed for the estimation of free fatty acids in the mammary gland tissue. The methodology of FAME was elaborated elsewhere [25].

### Western blotting

The mammary gland tissue sample was homogenized using RIPA lysis buffer, isolated by acetone precipitation and total protein concentration was assayed by using Bradford method [26]. All the protein samples were resolved on SDS-PAGE gels with Tris buffer system. The proteins were transferred to PVDF membrane by electroblotting by semidry system. Prestained protein ladder was used as a molecular weight identification marker. After 2 h of blocking with blocking buffer at room temperature, membranes were processed for primary antibody binding (1: 2000 dilutions) against Bcl-xl (MA-5-15,142), BAD (SC-8044), NFκBp65 (MA5–1616), UCHL-1 (MA1–83428) and COX (MA5–14568) in 4 °C followed by respective secondary antibody (anti-rabbit (SC-2030), anti-goat (SC-2020) and anti-mouse (31,430, Pierce Thermo Scientific, USA) in 1:5000 dilutions. β-actin (MA5–15739-HRP) was used as loading control. The PVDF blotting membranes were processed at final steps by using an enhanced ECL substrate (Western Bright ECL HRP substrate, Advansta, Melanopark, California, US) in Geldock system. The proteins bands were quantified using ImageJ software [27].

### Digital image analysis

For microscopic analysis data obtained from H&E and SEM micrographs, six randomly chosen regions were selected from all group and analysed independently for each respective sample. To identify the luminescence from experimental images and convert them into the heights to the final surface plot,3D interactive surface plot experiments was used to identify the pure H&E stained and SEM areas nearest neighbouring sampling in a square plot. This unmixing ultimately helps to distinguish between nuclear, ECM and background stains in a single complimentary image. Automated score was assigned by observing and measuring the pure H&E staining pattern and SEM micrograph in Image J standard program feature.

### Protein–protein interaction and gene ontological studies

To identify the various functional relations between different gene and protein protein-protein interactions were studied using STRING 10.0. Different biological process, cellular component and biological functions has been identified by gene ontological study (Search tool for the Retrieval of Interacting Genes/Proteins) software (http://string-db.org).

### Statistical analysis

All data were presented as mean ± SD and analyzed by one way ANOVA followed by Bonferroni’s multiple comparison tests for the possible significance identification between the various groups *p < 0.05, **p < 0.01, ***p < 0.001 were considered statistically significant. Statistical analysis was carried out using Graph Pad Prism (5.01), San Diego, California.

## Results

### Effect of Tadalafil upon hemodynamic changes

**Fig. 1:**
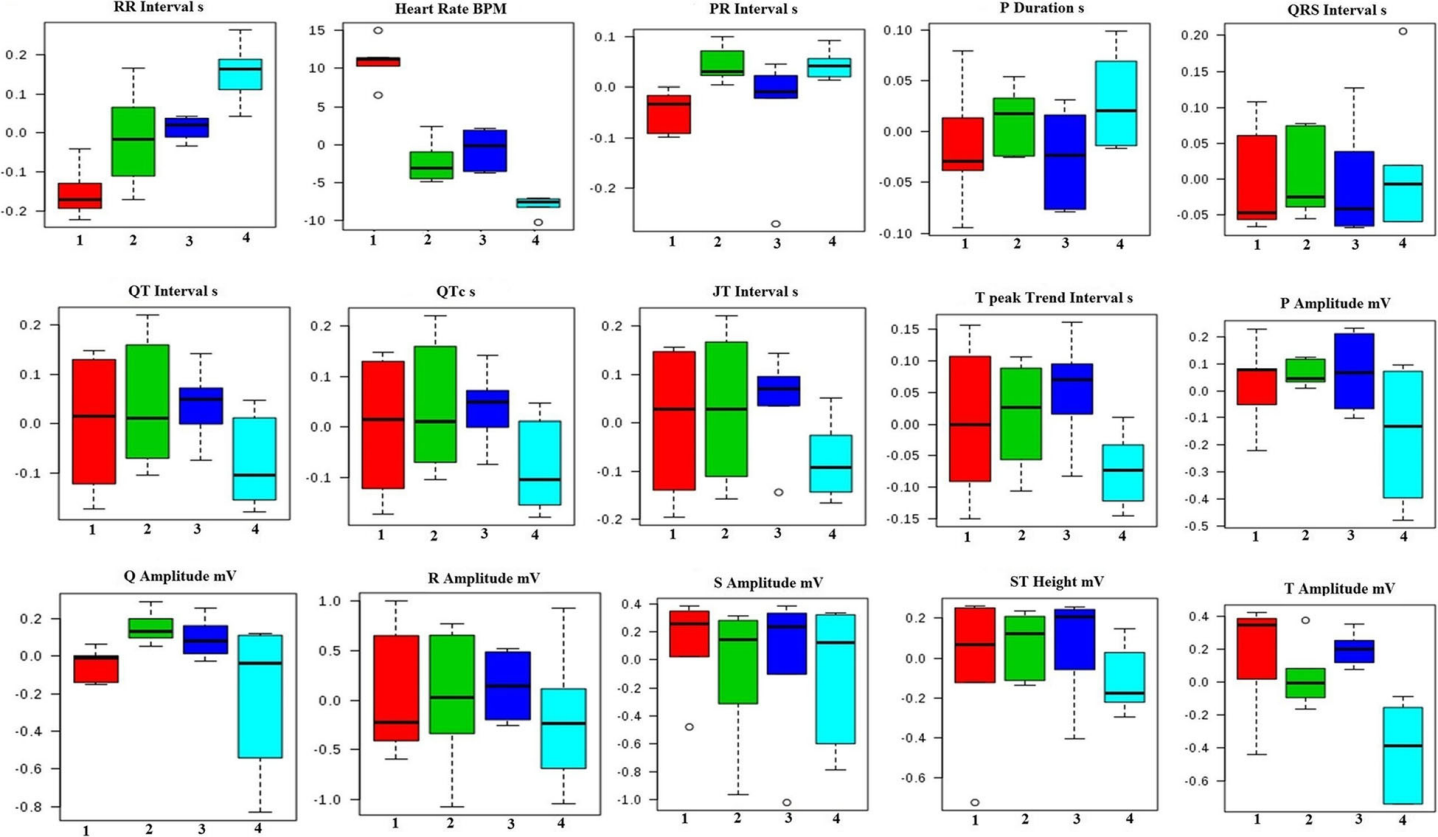
Effect of Tadalafil treatment on ECG recording. Representative box-cum-whisker plots showing quantitative variations of relative signal integrals for autonomic dysfunction relevant in the context of pathophysiology of mammary gland cancer. Groups were differentiated as: 1-Control (Normal saline, 3 ml/kg, p.o.), 2-Toxic control (MNU 47 mg/kg, i.v.), 3- MNU + Tadalafil (47 mg/kg i.v. + 2 mg/kg p.o.), 4- MNU + Tadalafil (47 mg/kg i.v. + 4 mg/kg p.o.). For presented ECG recordings, the VIP score > 1 and statistical significance is at the level of *p* ≤ 0.05. In the box plots, the boxes denote interquartile ranges, horizontal line inside the box denote the median, and bottom and top boundaries of boxes are 25^th^and 75th percentiles, respectively. Lower and upper whiskers are 5^th^and 95th percentiles, respectively

Heart rate variability (HRV) is the noninvasive measures of autonomic nervous system (ANS) regulation. It was previously reported that altered and dysfunctional ANS has been reported cancer patients. The RR interval was elevated in MNU treated group (0.18 ± 0.02) and Tadalafil in low dose (2 mg/kg, p.o.) restored RR interval about to normal (0.16 ± 0.01). On the other hand, heart rate (HR) was increased in MNU treated group (331.95 ± 0.68) which was diminished after Tadalafil treatment in dose dependent manner. QT interval was considerably declined in toxic control group (0.05 ± 0.01) which was restored after Tadalafil treatment (0.06 ± 0.01) (Fig. 1).

**Fig. 2:**
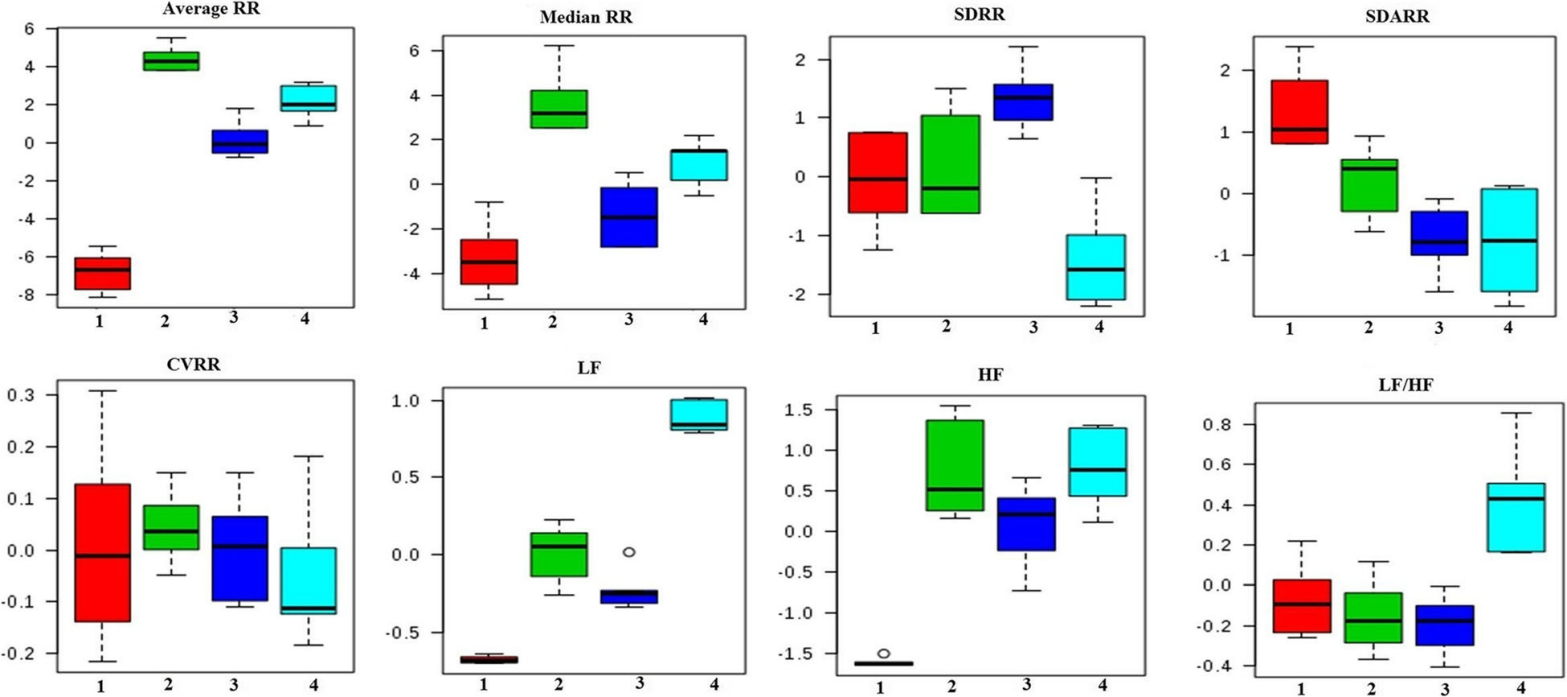
Effects of Tadalafil treatment on HRV. Representative box-cum-whisker plots showing quantitative variations of relative signal integrals for HRV parameters relevant in the context of pathophysiology of mammary gland cancer. Groups were differentiated as: 1-Control (Normal saline, 3 ml/kg, p.o.), 2-Toxic control (MNU 47 mg/kg, i.v.), 3- MNU + Tadalafil (47 mg/kg i.v. + 2 mg/kg p.o.), 4- MNU + Tadalafil (47 mg/kg i.v. + 4 mg/kg p.o.). For presented heart rate variability, the VIP score > 1 and statistical significance is at the level of p ≤ 0.05. In the box plots, the boxes denote interquartile ranges, horizontal line inside the box denote the median, and bottom and top boundaries of boxes are 25^th^and 75th percentiles, respectively. Lower and upper whiskers are 5^th^and 95th percentiles, respectively

In accordance with HRV parameters, the frequency domain parameters like average RR, median RR, SDRR and SD rate were elevated after MNU treatment. Tadalafil treatment helped to restore the above said parameters about to normal (Fig. 2).

### Morphological analysis of rat mammary gland tissue

#### Carmine staining

**Fig. 3:**
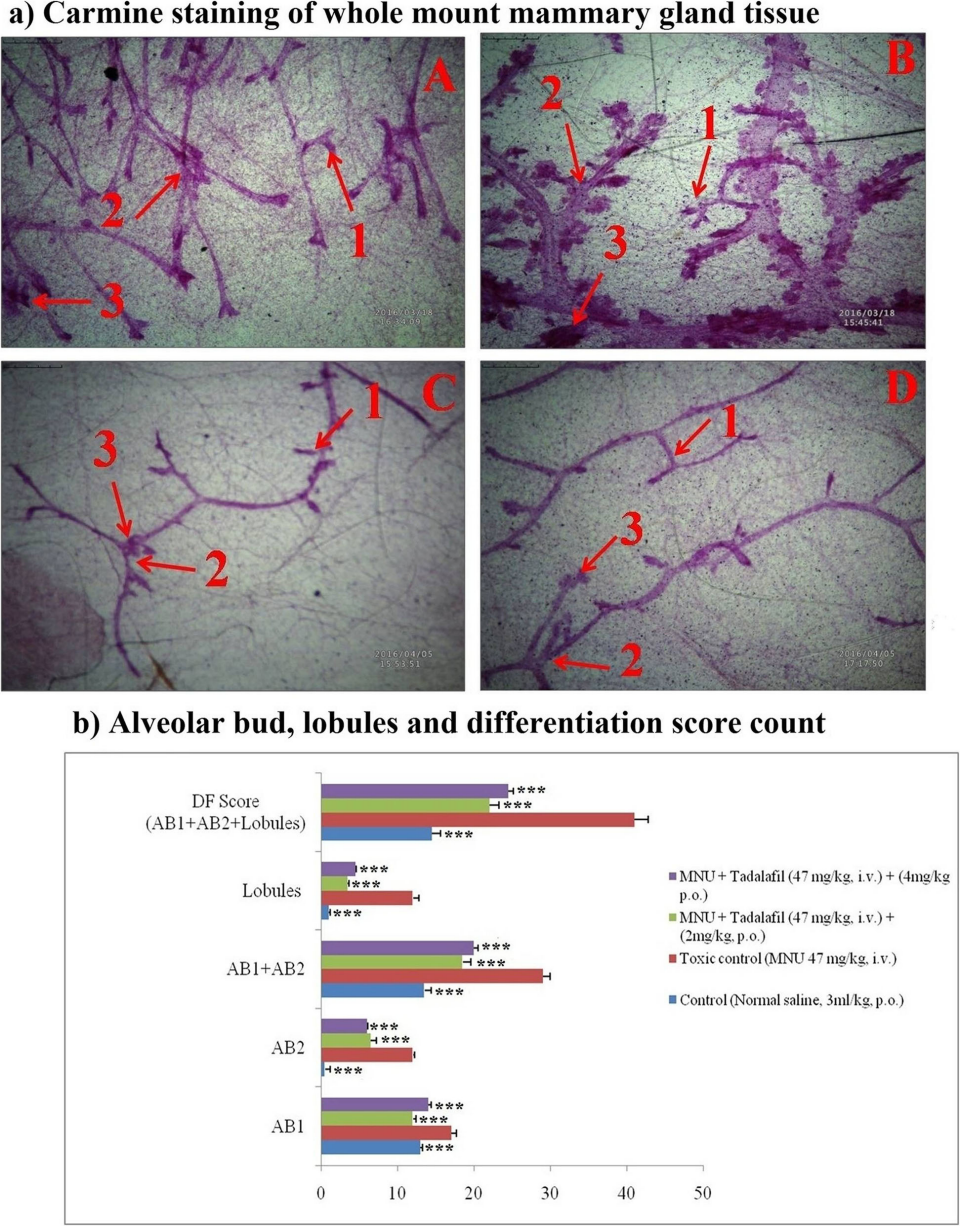
Carmine staining of mammary gland whole mount tissue. Whole mount carmine alum staining of ductal epithelium reveals the presence of AB1 (1), AB2 (2) and lobules (3) in respective groups [**a**-control (normal saline, 3 ml/kg, p.o.); **b**-toxic control (MNU 47 mg/kg, i.v.); **c** (MNU + Tadalafil; 47 mg/kg i.v. + 2 mg/kg p.o.) and **d** (MNU + Tadalafil; 47 mg/kg i.v. + 4 mg/kg p.o.)]. The extent of AB and lobules formation is excessive in the toxic group (**b**) which was subsided after Tadalafil treatment (**c** & **d**). The extent of AB and lobules formation is excessive in the toxic group (**b**) which has been subsided with respective treatment groups (**c** & **d**). The ABs score, lobules and DF scores were also higher in toxic control which was subsided after Tadalafil treatment in a dose dependent manner

The two main markers for angiogenesis are alveolar bud (ABs) count and differentiation (DF) score. In carmine staining, the no. of ABs and DF score were calculated. The ABs (both AB1 and AB2), lobules and DF were elevated after MNU treatment (17 ± 5.65, 12 ± 5.65, 12 ± 2.82 and 41 ± 14.12) and after Tadalafil treatment; all the parameters of cell proliferation were restored to normal (Fig. 3a-d).

#### H&E staining

**Fig. 4:**
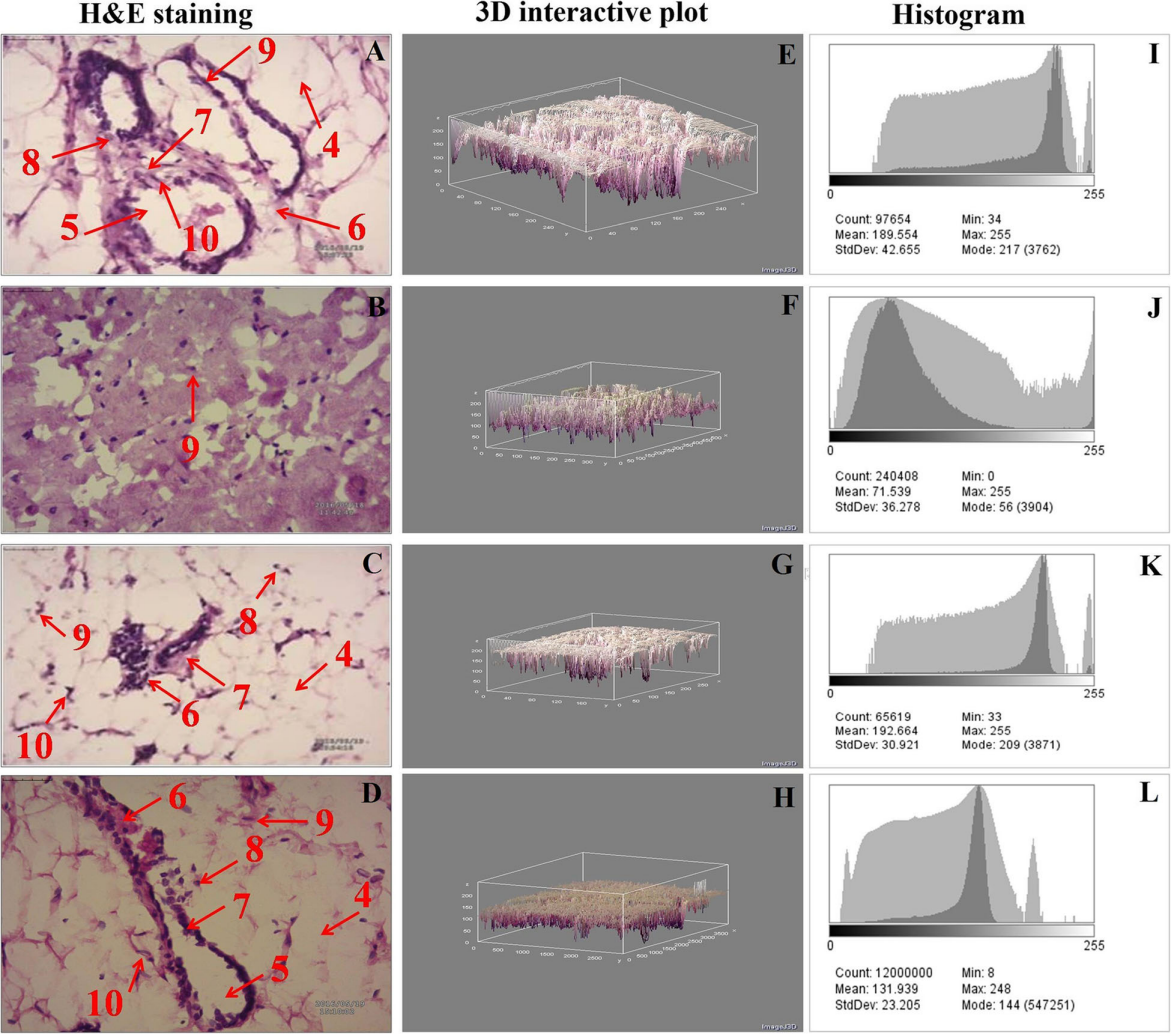
H&E analysis and digital image analysis of mammary gland tissue. H&E staining of four individual groups [**a**-control (normal saline, 3 ml/kg, p.o.); **b**-toxic control (MNU 47 mg/kg, i.v.); **c** (MNU + Tadalafil; 47 mg/kg i.v. + 2 mg/kg p.o.) and **d** (MNU + Tadalafil; 47 mg/kg i.v. + 4 mg/kg p.o.)] revealed presence of adipocytes (4), duct (5) LCT (6), DCT (7), lymphocytes (8), CEC (9) and MEC (10) in control as well as Tadalafil treated groups (**a**, **c** and **d**). In the toxic control group (**b**), the cell morphology was distorted, and cell organelles were absent. 3D image reconstruction and software-based analysis dataset of constructs representing score was done by using Image J (NIH) software by thresh holding of stained zones of H&E images followed by pixel vs intensity determination by the 3D interactive surface plot and log-histogram analysis (**e**,**f**,**g**,**h** and **i**,**j**,**k**,**l**).

H&E staining provide the cellular and morphological alterations occur in the tissue. In normal control tissues, all the cell organelles were clearly visible (Fig. 4a). MNU treatment was evident in scattered pattern of cuboidal epithelial cells (CEC), myoepithelial cells (MEC), lymphocytes, adipocytes and duct along with loose connective tissue (LCT) and dense connective tissues (DCT) (Fig. 4b). Tadalafil treatment restored all the parameters in dose dependent manner (Fig. 4c and d).

#### SEM analysis

**Fig. 5:**
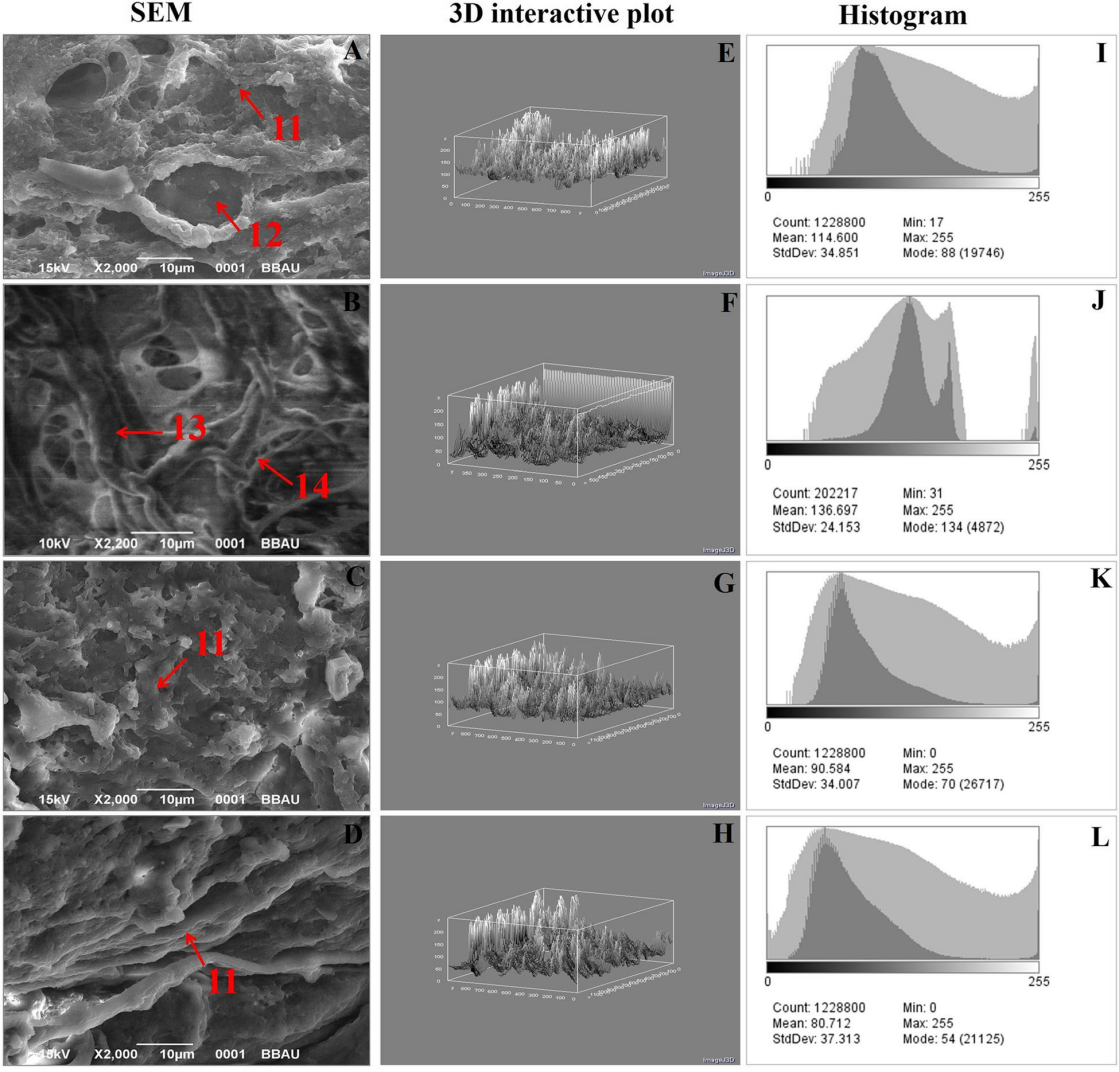
SEM and digital image analysis of mammary gland tissue. SEM analysis of four individual groups [**a**-control (normal saline, 3 ml/kg, p.o.); **b**-toxic control (MNU 47 mg/kg, i.v.); **c** (MNU + Tadalafil; 47 mg/kg i.v. + 2 mg/kg p.o.) and **d** (MNU + Tadalafil; 47 mg/kg i.v. + 4 mg/kg p.o.)] was performed. Control (**a**) demonstrated presence of collagenous layers (11), duct (12), nodules (13) and small capillary network (14). MNU (**b**) administration perceived loss of collagenous covering (11) and formation of tumormicrovessels/small capillary network (14) and nodules (13). Tadalafil treatment restores all the cell organelles close to normal. 3D image reconstruction and software-based analysis dataset of constructs representing score was done by using Image J (NIH) software by thresh holding of stained zones of H&E images followed by pixel vs intensity determination by the 3D interactive surface plot and log-histogram analysis (**e**,**f**,**g**,**h** and **i**,**j**,**k**,**l**)

SEM analysis was used to analyse the internal surface of whole cells and its organelles. In normal control tissue, collagen layer, duct and intra-arterial cushion/collagenous covering, were seen clearly (Fig. 5a). All the cellular organelles were absent in MNU treated group (Fig. 5b). Tadalafil treatment perceived decrease in tumor micro-vessel formation with restoration of intra-arterial cushion (Fig. 5c and d).

### Effect of antioxidant markers upon mammary gland carcinoma

**Fig. 6:**
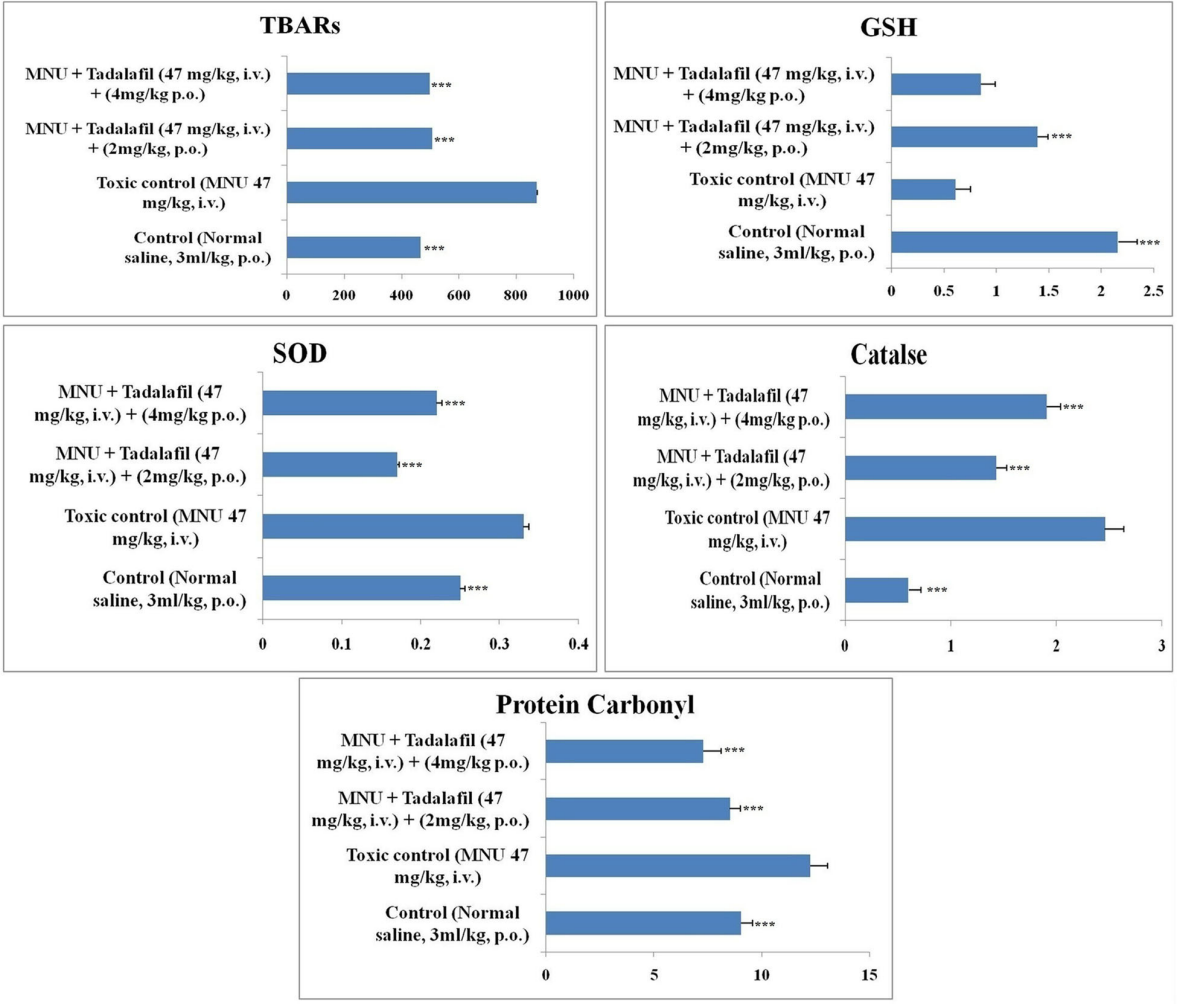
Effect of Tadalafil on the antioxidant markers. Bar diagram represents the difference between the control, toxic and Tadalafil treated groups. The markers of protein and lipid (PC and TBARs) were increased in MNU treated group and reduced after Tadalafil treatment. GSH was decreased after MNU treatment whereas the level of SOD and catalase were increased after MNU treatment which was subsided after Tadalafil treatment. All the data was presented as mean ± SD. Comparisons were made on the basis of one-way ANOVA followed by Bonferroni multiple test and all groups are compared to the toxic control group (*p < 0.05, **p < 0.01, ***p < 0.001)

Reactive oxygen species (ROS) are the byproducts of normal metabolism through the electron transport chain. ROS and other oxidative stress are considered as a driving force for cancer progression. Tadalafil treatment helps to restore the diminished antioxidant profile, when compared with MNU treated toxic group. The phenomena of protein and lipid peroxidation were very well evident after MNU treatment which correlates with the high level of TBARs and PC after MNU treatment (872.12 ± 72.98 nM of MDA/μg of protein and 12.24 ± 1.79 nM/ml respectively). Tadalafil treatment decreased the level of TBARs and PC in dose dependent manner (499.79 ± 109.2298 nM of MDA/μg of protein and 7.28 ± 0.82 nM/ml). GSH level in MNU treated group (0.61 ± 0.04 mg %) was restored to almost control group after Tadalafil treatment (1.39 ± 0.10 mg %). The level of SOD and catalase was increased after MNU treatment (0.33 ± 0.07 units of SOD/mg of protein and 2.46 ± 0.48 nM of H_2_O_2_/min/mg of protein) which was decreased after Tadalafil treatment to a significant level (i.e. 0.17 ± 0.13 units of SOD and 1.43 ± 0.01 nM of H_2_O_2_) (Fig. 6).

### Effect of inflammatory markers upon mammary gland carcinoma

**Fig. 7:**
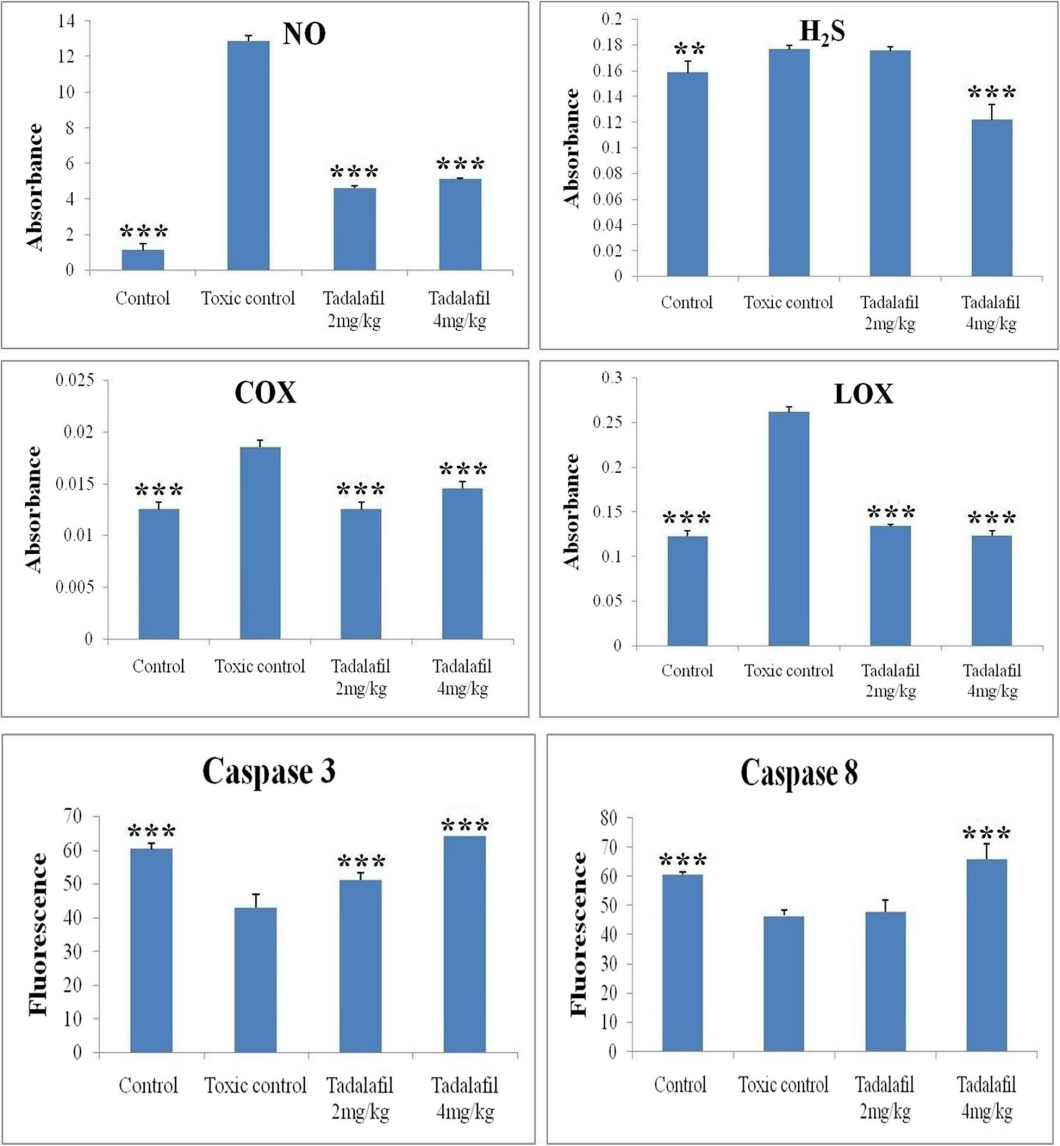
Effect of Tadalafil upon various inflammatory and apoptotic markers. Groups are defined as: Control (Normal saline, 3 ml/kg, p.o.); Toxic control (MNU 47 mg/kg, i.v.); MNU + Tadalafil (47 mg/kg i.v. + 2 mg/kg p.o.) and MNU + Tadalafil (47 mg/kg i.v. + 4 mg/kg p.o.).Values were expressed as mean ± SD. Comparisons were made on the basis of one-way ANOVA followed by Bonferroni multiple test and all groups are compared to the toxic control group (*p < 0.05, **p < 0.01, ***p < 0.001)

Inflammatory markers produced reactive nitrogen species (RNS) and ROS from epithelial and inflammatory cells, which leads to DNA damage. The inflammatory markers (NO, H_2_S, COX and LOX) were upregulated after MNU treatment. After Tadalafil treatment, the level of all the inflammatory markers was downregulated (Fig. 7).

### Effect of Tadalafil upon caspase assay

Caspases cysteinyl protease family members and they play a crucial role in apoptosis. The serum caspase 3 and caspase 8 was measured using fluorometric assay kits. The caspase 3 and caspase 8 levels were decreased in MNU treated group and after Tadalafil treatment, the levels of casapse 3 and caspase 8 were upregulated in a dose dependent manner (Fig. 7).

### Effect of Tadalafil upon FAME analysis

FAME assay converts cellular lipids into fatty acid methyl esters, resolved and identified by gas chromatography. After MNU treatment, there was substantial increase in saturated fatty acid, monounsaturated fatty acid along with decrease in the unsaturated fatty acid and polyunsaturated fatty acid content. Tadalafil treatment restored the lipid content in MNU treated animals (Additional file 1: Figure S1 and Additional file 2: Table S1).

### Immunoblotting analysis

**Fig. 8:**
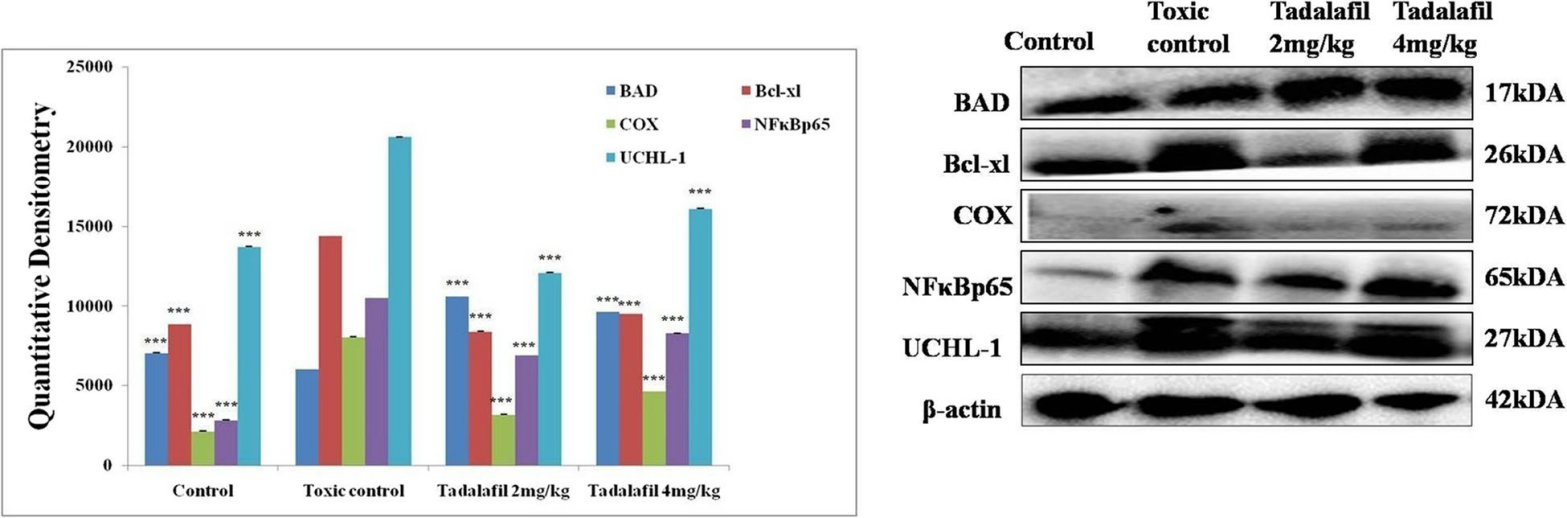
Tadalafil mediated activation of mitochondrial mediated apoptosis pathway. Protein extracted from individual groups [Control (Normal saline, 3 ml/kg, p.o.); Toxic control (MNU 47 mg/kg, i.v.); MNU + Tadalafil (47 mg/kg i.v. + 2 mg/kg p.o.) and MNU + Tadalafil (47 mg/kg i.v. + 4 mg/kg p.o.)] were subjected to immunoblotting of proapoptotic (BAD) and anti-apoptotic (Bcl-xl) marker along with COX, NFκBp65 and UCHL-1. β-actin was used as internal loading control. Each experiment was performed in triplicate. The data was represented as mean ± SD. The groups were significantly different by one-way ANOVA followed by Bonferroni multiple tests. All groups were compared to the toxic control group (*p < 0.05, **p < 0.01, ***p < 0.001)

Western blotting is used to separate and identify proteins. In this technique a mixture of proteins is separated on the basis of molecular weight through gel electrophoresis. Bcl-xl (anti-apoptotic) expression was increased after MNU treatment with diminished level of BAD (proapoptotic). Tadalafil treatment helped to restore the same and suggesting mitochondrial dysfunction (Fig. 8). MNU treatment validates the inflammation when perceived through increased expression of COX, NFκBp65 and UCHL-1. Tadalafil treatment dose-dependently curtails down the expression of COX, NFκBp65 and UCHL-1(Fig. 8).

## Discussion

Despite vast development in the cumulative survival rate of hormone dependent breast cancer patients but post-operative or chemotherapy received advanced metastatic stage still a positive life-threatening for cancer patient. Most primitive challenges in mammary gland carcinoma are to target different key proteomic marker that plays a crucial role in downstream pathways of carcinogenesis and distinct tissue metastasis. Thus it will be worthy to identify the proteomic check point for the development of innovative and safe therapeutic outcome to control and curing of malignancy. The present study demonstrated the repurposing effect of the PDE-5 inhibitor (Tadalafil) upon MNU induced mammary gland carcinoma in albino wistar rats. MNU is one of the classical laboratory carcinogens of alkyl-nitrosourea family which has the ability to directly alkylate DNA [28]. MNU is a widely used chemical carcinogenic model for induction of mammary gland carcinoma in albino wistar rats [14]. Cumulative cardiotoxicity and autonomic dysfunction is a well evident side effect of long term chemotherapy regimen of cancer patients [29]. It ultimately hinders the application of so many potent active chemotherapeutic agents. Being evidently well effective in pulmonary arterial hypertension, pulmonary vasodilatation, cardiac remodelling, diastolic function, interstitial fibrosis and inflammation, coronary microcirculation it was an urgent need to evaluate the cardiological parameter during the study and report safety of Tadalafil dose used throughout the study. Reduced HRV constitutes an independent prognostic factor for autonomic dysfunction. It was previously reported that HR was increased in breast cancer patients [30].RR interval, R waves and HR are the parameters which represent autonomic dysfunction. In the present study, the RR interval, R waves and HR were increased in MNU treated group which was decreased after Tadalafil treatment (Fig. 1). In the time domain, we analysed average RR, median RR, SDRR, CVRR and SD rate. All the cardiological parameters were significantly increased in MNU treated group and Tadalafil treatment helps normalised it. The reason to follow the main spectral components of LF is to analyse the frequency domain and measure sympathetic and parasympathetic activities. On the other hand, vagal parasympathetic activity and LF/HF ratio which indicates the sympathovagal balance also examined. In MNU treated group; LF, HF and LF/HF were increased which were subsided after Tadalafil treatment (Fig. 2). In summary, PDE-5 inhibitor selectively exhibit pleiotropic actions through pulmonary vasodilatation, LV remodelling, improved diastolic function, negative inotropic effects in naive cardiac myocytes, antiarrhythmic properties which ensure its long term safety in chemotherapeutic regimen.

The anti-carcinogenic activity of Tadalafil was again evaluated on the grounds of morphological architecture using carmine staining, H&E staining and SEM analysis. The results of carmine staining were in line with the previous reported results [14]. Increased ABs, lobules and DF score is the sign for cellular proliferation and the same was reported in MNU treated group [27]. The number of ABs, lobules and DF score were decreased after Tadalafil treatment in dose dependent manner (Fig. 3a-d). Histopathogical analysis of control group revealed the presence of CEC, MEC, lymphocytes, adipocytes, duct, LCT and DCT. All the cell organelles were distorted after MNU treatment, LCT and DCT were hard to identify. After Tadalafil treatment, all the cell organelles were restored in dose dependent manner (Fig. 4a-d). Mammary gland tissues were also evaluated for its surface texture analysis using SEM. After MNU treatment, marked proliferation was observed with increased in micro vessel formation, loss of intra-arterial cushion and vascular conglomeration. Tadalafil treatment restored the cellular architecture after SEM analysis (Fig. 5a-d). The finding from the morphological analysis revealed that Tadalafil in dose dependent manner restored the features of cellular proliferation and warrants its role as an anti-carcinogenic drug.

Oxidative stress is a well-known fact in cancer progression. It is defined as a physiological state in which the level of ROS is higher and generation of free radical is occurred. In mitochondria of the cell, ROS is generated through adenosine triphosphate (ATP) where electrons react with oxygen (O_2_) and results in the formation of superoxide anion (O_2_^.^) [31]. Downstream experimental condition reveals high level of NO in toxic group which may be endogenous NO which inhibited cytochrome c oxidase, causing generation of excess ROS. Previous studies validated that oxidative stress is linked with many of the pathophysiological diseases. Oxidative stress leads to damaging of the DNA molecule and regulates the progression of many cancers [32]. The markers of oxidative stress are TBARs, PC, SOD, catalase and GSH. In the present study, protein markers (TBARs and PC) were increased after MNU treatment and these markers decreased after Tadalafil treatment in dose dependent manner (Fig. 6). The level of GSH was decreased in MNU treated group which was restored after Tadalafil treatment. SOD and catalase are the major component of antioxidant defence system and the level of SOD and catalase was increased after MNU treatment which was normalised after Tadalafil treatment (Fig. 6).

Inflammatory markers also have a profound effect upon cancer prognosis. Tadalafil is a well-known specific inhibitor of the NO/cGMP pathway which has been perceived by NO [33]. Significant downregulation has been observed in the treatment groups which revealed Tadalafil mediated inhibition of NO synthesis in tissue and inhibition of intracellular ROS generation [34]. Overexpression of cystathionine-gamma-lyase (CSE), acystathionine-beta-synthase (CBS), and 3-mercaptopyruvate sulfurtransferase (3-MST) which ultimately leads to increased amounts of H_2_S, which augmented tumour growth and distinct metastasis by activating cellular bioenergetics, proliferative, migratory, and invasive signalling pathways [35]. Involvement of NO-H_2_S dual signalling is responsible for the curtailment of ABC transported mediated drug resistance and inhibition of carcinogenesis via c-GMP blockade [36]. Tadalafil treatment downregulates the level of NO and H_2_S in MNU treated animals (Fig. 7).

Dual inhibition strategies for COX and LOX in cancer are one of the established areas of research and our laboratory is very much focused in the specific area of interest. Previously, we have shown that dual inhibition (specific combinations of COX and LOX metabolites) has been considered for future best possible chemotherapeutic regime [24]. Tadalafil treated group shows the reduction of COX and LOX when examined through biochemical and immunoblotting assay in comparison with MNU treated group. Several research reports suggested that the expression of COX is associated with incidence of breast cancer. It includes large tumour size, positive axillary lymph node metastasis and HER2-positive tumour status [37]. Previous study on mice and rats that moderate to high COX expression is related to generation of mammary gland tumours. The result of present study also indicates the same (Fig. 7). The expression of COX in MNU treated group was increased which was subsided after Tadalafil treatment. Modulation of downstream metabolite like PGE2, TXA2 and TXB2 and LTA4, LTB4, LTC4, LTD4, LTE4 can exploit multidirectional signalling cascade to exert their anticancer effects and the same have been explored in the present study with wider prospects to enlighten the chemotherapeutic strategies by repurposing the mechanism of PDE-5 inhibitor. Dual inhibition ensures the alteration of AA metabolism in cancer cells and exhibits subsided inflammation, cell proliferation and neo-angiogenesis. Identification of specific subtype of COX and LOX responsible for apotogenic potential of Tadalafil is the future direction of our research [38].

To establish possible mechanisms of the anticancer effects via PDE-5 inhibition and role in caspase-dependent apoptosis and cell growth arrest we have checked the caspase group of protein. Caspase 3 and caspase 8 are key regulators of the apoptotic response in which caspase 3 is executioner caspase and caspase 8 is initiator caspase. Caspase 8 is activated through self-cleavage and after that it activates downstream effector caspase like caspase 3 which [39] may be linked to concomitant increases in regulation of downstream pathways through JNK, mitogen-activated protein kinase 1, decreased Wnt/β-catenin expression and down-regulation of cyclin D, inhibition kinases 1/2 (ERK1/2) and alterations in the regulation of p42/p44 (MAPK) and p21 pathways. In the present study, the results from caspase assay revealed that after MNU treatment the level of caspase 3 and 8 was decreased and the same was restored after Tadalafil treatment (Fig. 7).

FAME analysis revealed the participation of saturated fatty acids, monounsaturated fatty acid, unsaturated fatty acid and polyunsaturated fatty acids. It was reported that women’s consumed saturated fatty acid in diet have a higher risk of breast cancer and the same observations were found in the present study [40]. The sharp peaks of saturated fatty acid were seen in MNU group which was subsided after Tadalafil treatment. Monounsaturated fatty acid has both effects depending upon the source of food. In MNU treated group, the level of monounsaturated fatty was increased and the same was decreased after Tadalafil treatment. It was previously reported that unsaturated Trans fatty acid may increase the risk of invasive breast cancer and the same observations were found in FAME analysis after MNU treatment [41]. Various research reports validated the promoting as well as inhibitory effect of polyunsaturated fatty acid and mammary gland tumorigenesis [41]. In the resent piece of work, inhibitory effects of polyunsaturated fatty acids were seen after Tadalafil treatment (Additional file 1: Figure S1 and Additional file 2: Table S1).

**Fig. 9:**
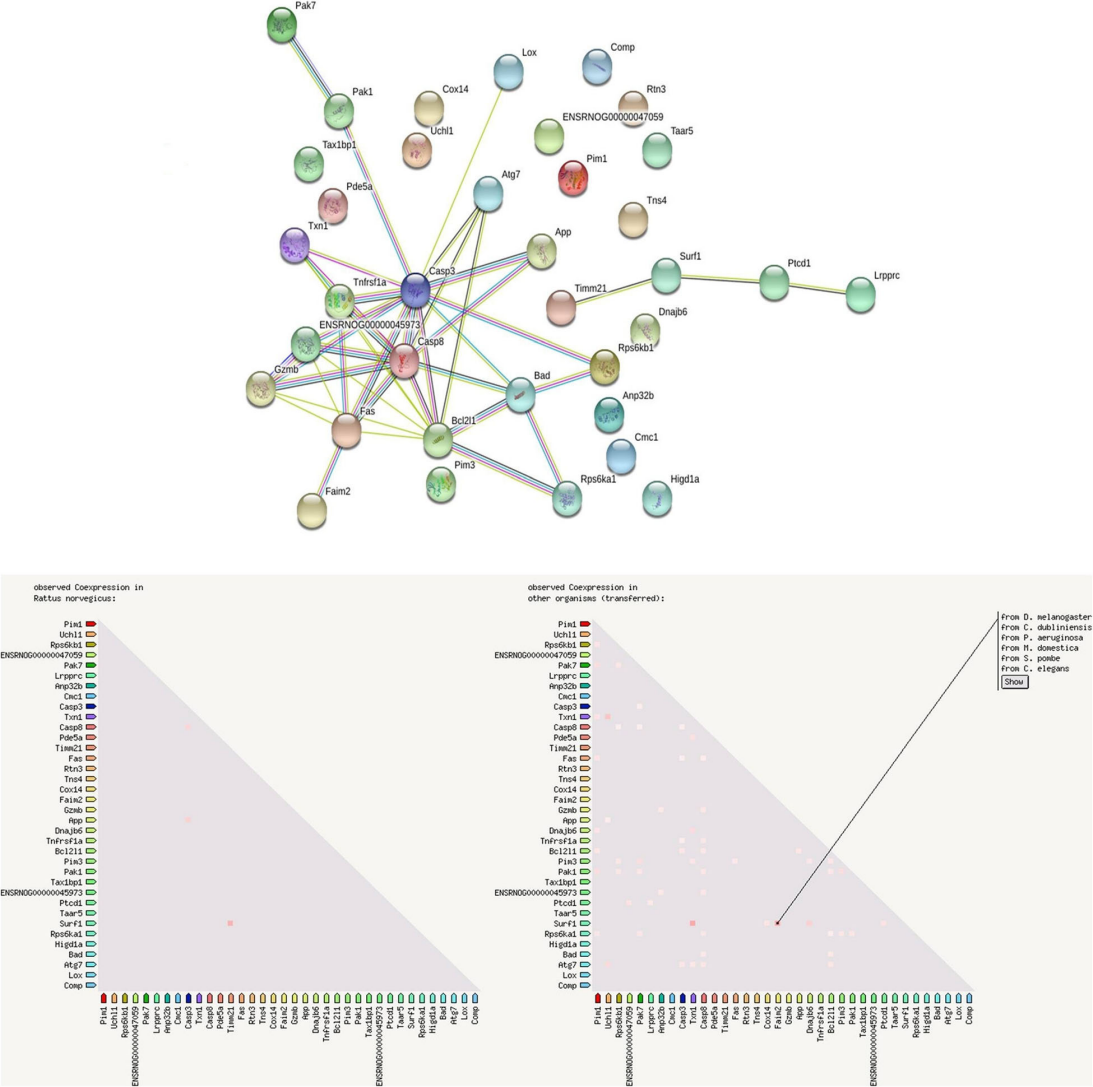
Protein-Protein interaction and Co-expression network analysis. Protein-protein interaction and co-expression analysis was performed using STRING V 10.0 with respect to differentially expressed proteins as identified from the Western blot analysis in relation to mitochondrial and hypoxic proteomic checkpoints

Cell apoptosis is a process of programmed cell death and it is regulated by two major pathways: intrinsic and extrinsic pathways. The intrinsic pathway and Mitochondrial Outer Membrane Permeabilization (MOMP) are regulated through the balance of pro- and anti-apoptotic proteins of Bcl-2 family [42, 43]. In the present study, the expression of anti-apoptotic protein (Bcl-xl) was increased after MNU treatment and the vice versa effect was found upon pro-apoptotic protein (BAD). Collaborative result from pro-apoptotic and anti-apoptotic protein with downstream caspase cascade validate the participation of Tadalafil in mitochondrial mediated death apoptosis pathway (Figs. 8 and 9).

Previous reports revealed that the 26S centre of UCHL-1 is regulated by 20S immunoproteasome subunit which inhibits the degradation of the ubiquitinated proteins [21]. In MNU treated group the expression of UCHL-1 was increased which was subsided after Tadalafil treatment. The same relation has been linked with the expression of NFκB/p50 precursor p105 and IκBα as it is essential for activity of NFκBp65. In line with to above results, the expression of NFκBp65 was increased in MNU treated group which was decreased after Tadalafil treatment (Figs. 8 and 9).

## Conclusion

Authors would like to conclude that PDE-5 inhibitor (Tadalafil) had a marked effect upon MNU induced mammary gland cancer. The mechanism of action of Tadalafil is validated through mitochondria mediated death apoptosis pathway and Tadalafil also decreased the inflammatory markers. Earlier, it was also reported that combination of sildenafil with celecoxib was found to be cytotoxic in breast, hepatoma, colorectal cancer, glioblastoma and medulloblastoma cell lines. It would be helpful if well directed future preclinical and clinical study targeting the repurposing effect of PDE5 inhibitor, to explore its additional mechanism. Tadalafil has low-cost and low-toxicity with great preclinical potential which can be further explored through well directed clinical trial in near future to include it with current and emerging standard of care treatments in breast cancer.

## Supplementary information

**Additional file 1: Figure S1.** FAME analysis of the mammary gland tissue subjected to MNU and Tadalafil.

**Additional file 2: Table S1.** Fatty acid profiling of mammary gland tissue treated with MNU and Tadalafil.

## Data Availability

The datasets used and analysed during the current study are available from the corresponding author on reasonable request.

## Abbreviations

3-MST: 3-mercaptopyruvate sulfurtransferase
AA: Arachidonic acid
ABs: Alveolar buds
ATP: Adinosine triphosphate
BSA: Bovine serum albumin
cAMP: Cyclic adenosine monophosphate
cGMP: Cyclic guanosine monophosphate
CBS: Acystathionine-beta-synthase
CEC: Cuboidal epithelial cell
COX: Cycloxygenase
CSE: Cystathionine-gamma-lyase
DCT: Dense connective tissue
DF: Differentiation score
DTT: Dithiothreitol
ECG: Electrocardiogram
ERK: Extracellular-signal regulated kinases
FAME: Fatty acid methyl esters
FTC: Ferrithiocyanate
GSH: Glutathione
H&E: Hematoxyline and eosin
H_2_S: Hydrogen sulphide
HBSS: Hank’s balanced salt solution
HR: Heart rate
HRV: Heart rate variability
LOX: Lipoxygenase
MAPK: Mitogen activated-protin kinase
MDSCs: Myeloid derived suppressor cells
MEC: Myoepithelail cell
MNU-n: Methyl-n-nitrosourea
MOMP: Mitochondrial membrane permeabilization
NO: Nitric oxide
PC: Protein carbonyl
PDE: Phosphodiesterase
ROS: Reactive oxygen species
SEM: Scanning electron microscopy
SOD: Superoxide dismutase
TBARs: Thiobarbituric acid reactive substances
TMPD- N,N,N′N′: Tetramethyl-p-phenylenediamine
