# Correction: Repurposing mechanistic insight of PDE-5 inhibitor in cancer chemoprevention through mitochondrial-oxidative stress intervention and blockade of DuCLOX signalling

**DOI:** 10.1186/s12885-022-09928-z

**Published:** 2022-07-27

**Authors:** Manjari Singh, Sweta Kasna, Subhadeep Roy, Sara Aldosary, Abdulaziz S. Saeedan, Mohd. Nazam Ansari, Gaurav Kaithwas

**Affiliations:** 1 Department of Pharmaceutical Sciences, Babasaheb Bhimrao Ambedkar University, (A Central University), Vidya Vihar, Raebareli road, Lucknow, UP 226 025 India; 2 Department of Pharmaceutical Sciences, King Faisal University, Al-Ahsa, Saudi Arabia; 3 Department of Pharmacology, College of Pharmacy, Prince Sattam Bin Abdulaziz University, Al-Kharj, Kingdom of Saudi Arabia

**Correction: BMC Cancer 19, 996 (2019)**

**https://doi.org/10.1186/s12885-019-6152-9**

Following publication of the original article [1], it was reported that there was an error in Fig. 5. The corrected Fig. 5 is supplied in this correction article.

**Fig. 5:**
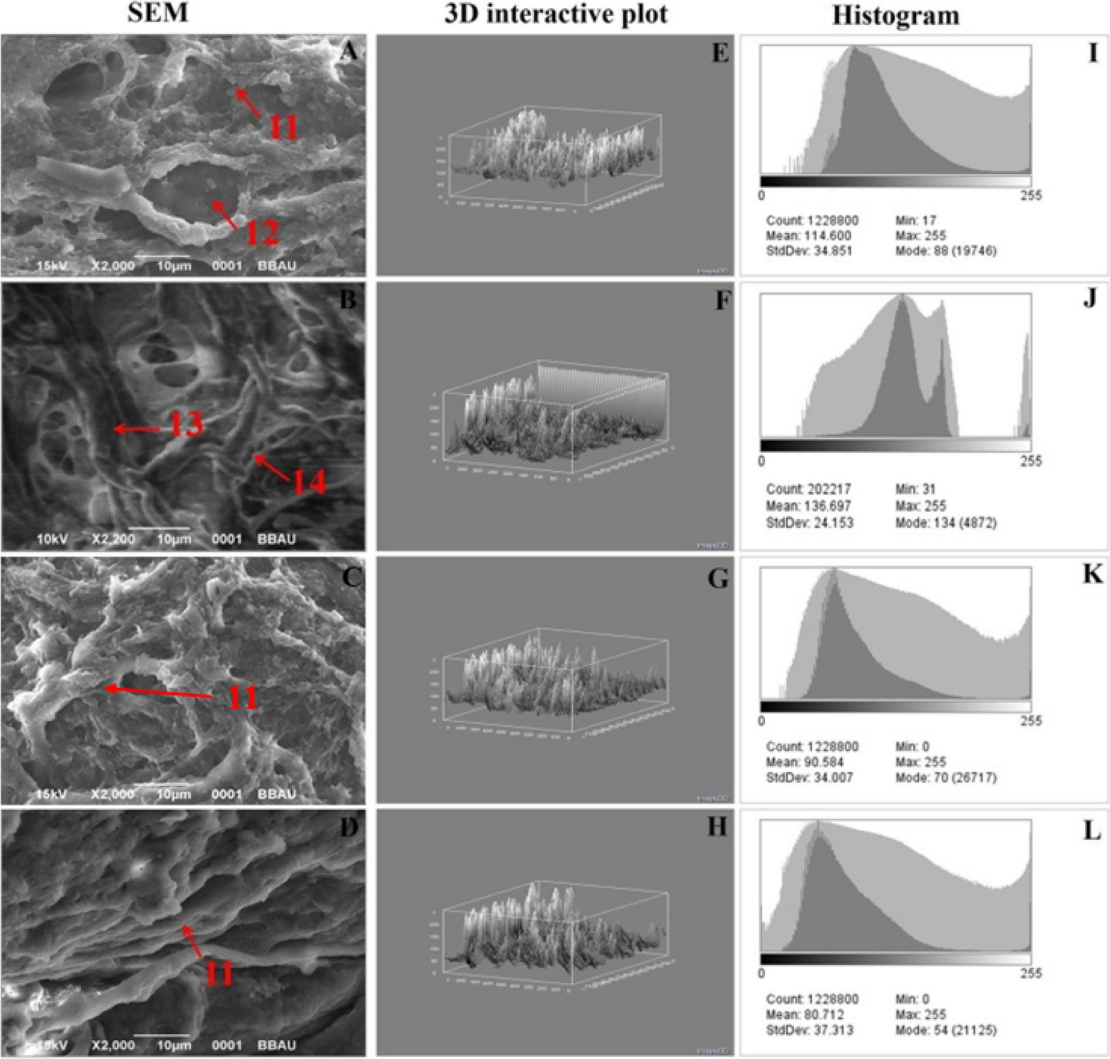
SEM and digital image analysis of mammary gland tissue. SEM analysis of four individual groups [**a**-control (normal saline, 3 ml/kg, p.o.); **b**-toxic control (MNU 47 mg/kg, i.v.); **c** (MNU + Tadalafil; 47 mg/kg i.v. + 2 mg/kg p.o.) and **d** (MNU + Tadalafil; 47 mg/kg i.v. + 4 mg/kg p.o.)] was performed. Control (**a**) demonstrated presence of collagenous layers (11), duct (12), nodules (13) and small capillary network (14). MNU (**b**) administration perceived loss of collagenous covering (11) and formation of tumormicrovessels/small capillary network (14) and nodules (13). Tadalafil treatment restores all the cell organelles close to normal. 3D image reconstruction and software-based analysis dataset of constructs representing score was done by using Image J (NIH) software by thresh holding of stained zones of H&E images followed by pixel vs intensity determination by the 3D interactive surface plot and log-histogram analysis (**e**,**f**,**g**,**h** and **i**,**j**,**k**,**l**)
