# Supplementary figures and images for: Repurposing mechanistic insight of PDE-5 inhibitor in cancer chemoprevention through mitochondrial-oxidative stress intervention and blockade of DuCLOX signalling
Source: BMC Cancer. 2019 Oct 24;19:996. doi: 10.1186/s12885-019-6152-9 (PMC6814136; doi:10.1186/s12885-019-6152-9)

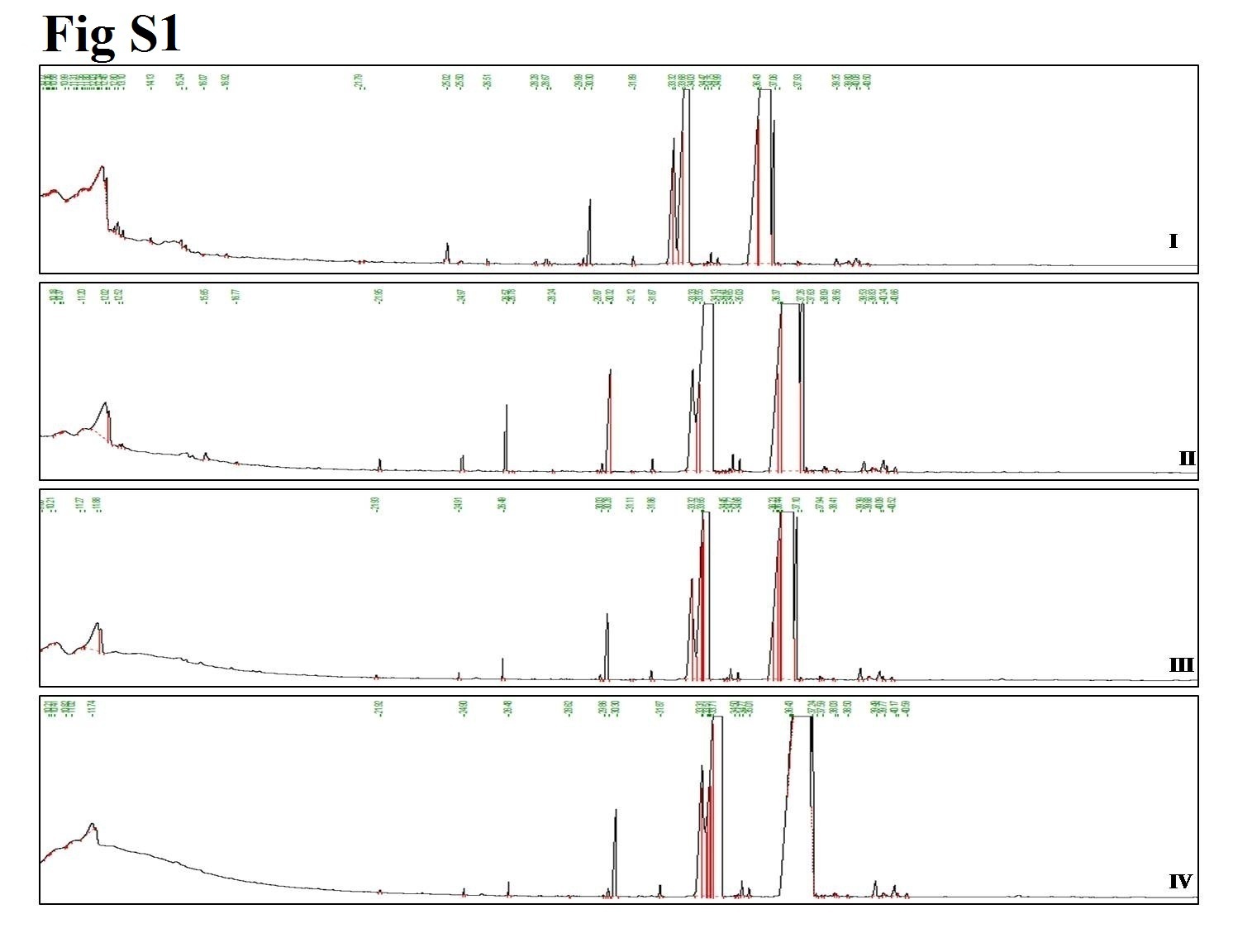

Supplement: Supplementary file 1 — Additional file 1: Figure S1. FAME analysis of the mammary gland tissue subjected to MNU and Tadalafil. [file 12885_2019_6152_MOESM1_ESM.jpg]
