# Supplementary material for: Repurposing mechanistic insight of PDE-5 inhibitor in cancer chemoprevention through mitochondrial-oxidative stress intervention and blockade of DuCLOX signalling
Source: BMC Cancer. 2019 Oct 24;19:996. doi: 10.1186/s12885-019-6152-9 (PMC6814136; doi:10.1186/s12885-019-6152-9)
Supplement: Supplementary file 2 — Additional file 2: Table S1. Fatty acid profiling of mammary gland tissue treated with MNU and Tadalafil. [file 12885_2019_6152_MOESM2_ESM.docx]

| **S. No** | **Name of Fatty Acids** | **Contro**l  (Normal saline,  3ml/kg p.o.) | **Toxic control**  (MNU, 47 mg/kg, i.v.) | **MNU + Tadalafil**  (47 mg/kg i.v.) +  (2 mg/kg p.o.) | **MNU + Tadalafil**  (47mg/kg i.v.) +  (4 mg/kg p.o.) |
| --- | --- | --- | --- | --- | --- |
| **Saturated fatty acid** | | | | | |
| 1 | Methyl Myristate (RT-25.02) | 0.15 | 0.69 | 0.14 | 0.10 |
| 2 | Methyl Pentadecanoate (RT- 30.30) | 0.72 | 1.15 | 0.80 | 1.39 |
| 3 | Cis-10-Pentadecenoic acid methyl (RT-31.89) | 0.06 | 0.09 | 0.07 | 0.10 |
| 4 | Methyl Palmitate (RT-33.32) | 1.27 | 0.37 | 1.19 | 1.89 |
| 5 | Methyl Heptadecanoate (RT-34.02) | 0.02 | 0.01 | 0.02 | 0.02 |
| 6 | Methyl stearate (RT-34.99) | 0.04 | 0.07 | 0.04 | 0.07 |
| 7 | Methyl Arachidate (RT-37.06) | 1.81 | 3.36 | 2.18 | 2.31 |
| **Monounsaturated fatty acid** | | | | | |
| 8 | Methyl Palmitoleate (RT-33.38) | 2.08 | 2.68 | 2.18 | 2.67 |
| 9 | Cis-9-Oleic Acid Methyl Ester (RT- 33.74) | 7.42 | 12.07 | 6.30 | 11.52 |
| 10 | Cis-10-Heptadecenoic acid methyl (RT-34.75) | 0.07 | 0.10 | 0.07 | 0.12 |
| 11 | Methyl cis-11-Eicosenoate (RT-39.35) | 0.08 | 0.13 | 0.14 | 0.24 |
| **Unsaturated trans fatty acid** | | | | | |
| 12 | Trans-9-Elaidic Methyl Ester (RT-33.67) | 2.17 | 1.13 | 1.03 | 2.88 |
| **Polyunsaturated fatty acid** | | | | | |
| 13 | Methyl Linoleate (RT-36.43) | 13.77 | 17.08 | 12.74 | 0.09 |
| 14 | Methyl Linolenate (RT-40.08) | 0.09 | 0.16 | 0.11 | 0.17 |

**Table S1: Fatty acid profiling of mammary gland tissue treated with MNU and Tadalafil**
